# Reliability and Validity of AI-Based Pose Estimation Algorithms for Assessing Lower-Limb Flexibility and Joint Range of Motion

**DOI:** 10.3390/s26185890

**Published:** 2026-09-17

**Authors:** Hinata Okuno, Tomoya Ishida, Yuta Koshino, Satoshi Kasahara, Harukazu Tohyama, Mina Samukawa

**Affiliations:** 1 Graduate School of Health Sciences, Hokkaido University, Kita-12 Nishi-5, Kita-ku, Sapporo 060-0812, Hokkaido, Japan; hinata.okuno9@gmail.com; 2 Department of Rehabilitation Science, Faculty of Health Sciences, Hokkaido University, Sapporo 060-0812, Hokkaido, Japan; t.ishida@hs.hokudai.ac.jp (T.I.); y-t-1-6@hs.hokudai.ac.jp (Y.K.); kasahara@hs.hokudai.ac.jp (S.K.); 3 Department of Orthopaedic Sports Science, Faculty of Health Sciences, Hokkaido University, Sapporo 060-0812, Hokkaido, Japan; tohyama@med.hokudai.ac.jp

**Keywords:** artificial intelligence, human pose estimation, markerless, human detection, muscle flexibility, joint range of motion

## Abstract

**Highlights:**

**What are the main findings?**

- AI-based pose estimation models (MediaPipe, OpenPose, and HRNet) showed high intra-rater reliability (ICC ≥ 0.90) and high concurrent validity (r^2^ = 0.73–0.94), comparable to conventional manual methods, for lower-limb flexibility and joint range of motion (ROM) tests: the Active Knee Extension Test (AKET), Modified Thomas Test (MTT), and Weight-Bearing Lunge Test (WBLT).
- Systematic biases with relatively large 95% limits of agreement (LOA) were observed in the AI-based pose estimation algorithms, indicating that the absolute values are not directly interchangeable with conventional measurements.

**What are the implications of the main findings?**

- AI-based pose estimation can serve as a scalable alternative assessment tool in settings where conventional manual measurements are impractical, such as large-scale screening and self-monitoring.
- AI-derived measurements should not be directly substituted for conventional reference values or cutoffs, highlighting the need to establish AI-specific reference values in future studies.

**Abstract:**

Muscle flexibility and joint range of motion (ROM) evaluation are essential for injury prevention and rehabilitation; however, conventional manual methods are limited in large-scale screening and self-monitoring because of examiner dependency and time constraints. This study evaluated the reliability and validity of AI-based pose estimation for lower-limb flexibility and ROM tests compared with conventional manual measurements. Twenty healthy volunteers (40 lower limbs; age 24.4 ± 2.6 years) underwent three flexibility tests—the Active Knee Extension Test (AKET), Modified Thomas Test (MTT), and Weight-Bearing Lunge Test (WBLT). The tests were assessed by a physiotherapist and three pose-estimation models (MediaPipe, OpenPose, and HRNet). Intra-rater reliability was evaluated with ICC(3,1). Concurrent validity and agreement were assessed using linear regression and Bland–Altman analysis. Intra-rater reliability was good to excellent for all methods (ICC ≥ 0.87), and concurrent validity was high (r^2^ ≥ 0.80), except for MTT using the HRNet (r^2^ = 0.73). Fixed biases were observed during the flexibility test. The limits of agreement ranged from ±8.04–10.87 cm for the AKET and MTT, and ±3.13–4.42 cm for WBLT. The WBLT showed a significant systematic bias, with an underestimation of −6.25° to −7.17° across all models. In young, healthy volunteers under standardized laboratory conditions, pose estimation models showed robust reliability and concurrent validity as objective tools for lower-limb flexibility assessment. However, systematic biases warrant caution regarding the interchangeability between AI-based measurements and conventional methods.

## 1. Introduction

The assessment of muscle flexibility and joint ROM is an important clinical measure for rehabilitation and injury prevention in the field of sports medicine. Specifically, tightness in the hamstring and rectus femoris muscles and limited ankle dorsiflexion ROM are associated with the risk of various sports injuries [1,2,3,4,5], requiring accurate and frequent assessments. To evaluate muscle flexibility and joint ROM, the Active Knee Extension Test (AKET), Modified Thomas Test (MTT), and Weight-bearing Lunge Test (WBLT) have been commonly employed. However, manual measurements using conventional tools, such as goniometers and inclinometers, require the identification of bony landmarks, which leads to examiner dependency and requires significant time and effort to perform. Therefore, applying these methods to large-scale screenings in sports settings is challenging.

In recent years, deep-learning-based pose estimation algorithms have emerged, enabling the automatic estimation of joint positions from images and videos captured using smartphones or monocular cameras [6,7,8]. These technologies are highly useful in clinical and sports settings because they are low-cost and easy to operate. Furthermore, their non-contact nature reduces the need for manual identification of anatomical landmarks [8]. This accessibility not only facilitates efficient evaluations by practitioners but also allows athletes to perform self-assessments in their homes or training settings. This convenience is particularly advantageous for continuous monitoring, large-scale screening, and tele-rehabilitation in areas with limited medical access.

Pose estimation models should be carefully applied, considering task dependency and potential inaccuracies in specific movements. During walking, OpenPose showed relatively good mean absolute errors ranging from approximately 2.3° to 5.1° for hip and knee angles relative to 3D marker-based motion capture [9,10] although accuracy varied with the pose estimation model from 3.7° to 9.4° across joints [10]. However, the measurement errors tend to increase under certain movements and conditions. For example, sagittal-plane motions are susceptible to self-occlusion, in which overlapping body segments obscure anatomical landmarks. A systematic review reported that errors can reach up to 28.6° in the sagittal plane [11], whereas they remain relatively low (0–8.3°) in the frontal plane. Moreover, movement-specific characteristics further contribute to accuracy; deep flexion, for instance, has been shown to exhibit reduced validity and increased estimation error. While knee joint angle estimation has generally shown relatively stable accuracy in previous studies (ICC = 0.83), hip joint angle estimation during bilateral squats demonstrated only fair validity (ICC = 0.37) and was affected by proportional bias, particularly as the flexion angle increased [12]. Therefore, additional validation is warranted when applying pose-estimation models to lower-limb flexibility and ROM assessments involving test-specific postures at the end range of motion.

Although pose estimation has emerged as a promising tool in sports science, no study has investigated the reliability and validity of AI pose estimation models compared with conventional manual methods for lower-limb flexibility and ROM tests: AKET, MTT, and WBLT. Because flexibility and ROM tests often involve test-specific postures with extreme joint positions, pose estimation accuracy may be compromised. Therefore, the first aim of this study was to evaluate the reliability and validity of pose-estimation models compared to conventional manual measurements in the Active Knee Extension Test (AKET) [13], Modified Thomas Test (MTT) [14,15], and Weight-Bearing Lunge Test (WBLT) [16]. The second aim was to compare the reliability, concurrent validity, and agreement of different pose estimation algorithms to determine the most effective model for each flexibility test. We selected MediaPipe, OpenPose, and HRNet because they represent distinct strategies: lightweight regression [17], bottom-up part association [18], and top-down high-resolution representation [19]. We hypothesized that pose-estimation models would show acceptable reliability and validity for lower-limb flexibility and ROM assessment, although their performance and agreement with conventional methods may vary across different test types.

## 2. Materials and Methods

### 2.1. Study Design

**Figure 1:**
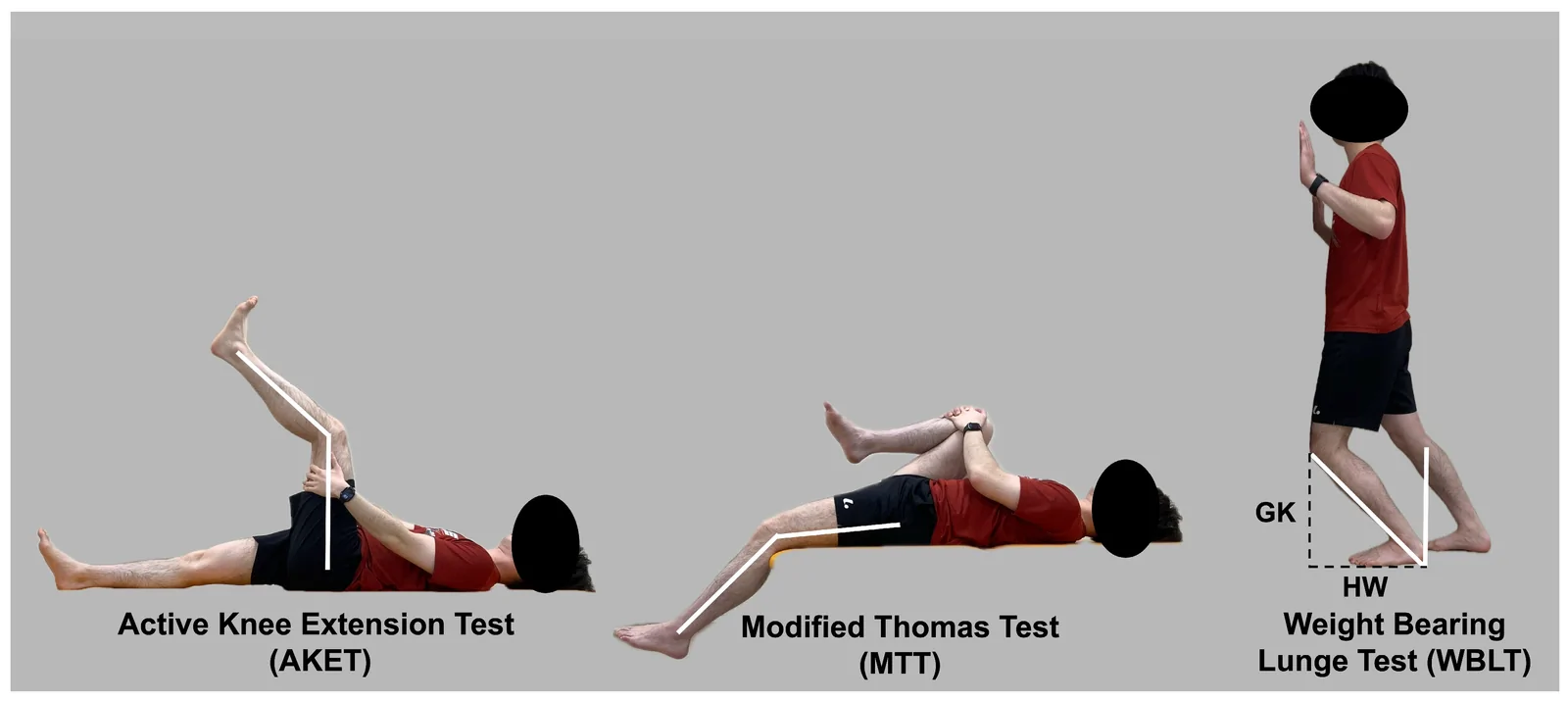
Representative positions of the lower-limb flexibility and ROM tests: active knee extension test (AKET), weight-bearing lunge test (WBLT), and modified Thomas test (MTT). The joint angles between the white lines were measured using a goniometer and the ImageJ software (1.54g). For the WBLT, the ankle dorsiflexion angle was assessed using a trigonometric method: 90° − arctan (GK/HW). GK: ground–knee distance; HW: heel–wall distance.

This study employed three flexibility and ROM measurements: AKET [13], MTT [14] and WBLT [20]. The participants wore T-shirts and shorts. While T-shirts and shorts were standardized, no restrictions were imposed on the color or printed designs, considering their clinical setting. Initially, the participants warmed up on a stationary ergometer for 5 min at a comfortable pace to standardize their activity. Subsequently, the participants performed each stretching protocol twice for 30 s, followed by a three-minute rest period (Figure 1). The order of the three flexibility measurements was fixed (AKET → MTT → WBLT), with a one-minute rest period between each test. In contrast, the measured body side (left or right) and the order of the conventional measurement and image capture (used for AI pose-estimation and ImageJ analysis) were randomized for each assessment. Each flexibility test was performed four times, and the first measurement was excluded from the analysis to account for any preconditioning effect.

### 2.2. Participants

A total of 20 healthy volunteers (10 males and 10 females; age, 24.4 ± 2.6 years; height, 165.7 ± 9.3 cm; weight, 57.9 ± 10.3 kg) were recruited for this study. The sample size of 40 limbs from 20 participants was determined based on a priori power analysis for reliability (ICC) and concurrent validity. For the reliability analysis, a sample size of *n* = 37 was required (expected ICC = 0.85, minimum acceptable ICC = 0.70, three trials, α = 0.05, power = 0.80). To evaluate concurrent validity, a minimum of *n* = 8 was required, assuming a coefficient of determination, r^2^ = 0.75 based on previous studies (α = 0.05, power = 0.80). Conversely, an a priori calculation was not performed for the Bland–Altman agreement analysis, and the sample size was determined according to previous research in the field [21,22]. To evaluate concurrent validity, a minimum of *n* = 8 was required, assuming a coefficient of determination, r^2^ = 0.75 based on previous studies (α = 0.05, power = 0.80). Conversely, an a priori calculation was not performed for the Bland–Altman agreement analysis, and the sample size was determined according to previous research in the field [21,22]. Participants who met the following criteria were included: individuals who were free from any neurological or orthopedic conditions in the target joints within the last six months that would impede the full range of motion. All participants were thoroughly informed of the procedures and potential risks involved and signed a written informed consent form before participation. The study protocol was approved by the Institutional Review Board of the Faculty of Health Sciences, Hokkaido University (approval number: [22,23,24,25]).

The measurements were performed in a laboratory maintained at a constant ambient temperature of 25 °C. The illumination comprised room light and minimal natural light against a plain white wall. A tablet (iPad Pro 11-inch, 2nd generation, Apple Inc., Cupertino, CA, USA) was used as the camera and was mounted on a tripod. The images were captured using the rear 12-megapixel camera at a resolution of 4032 × 3024 pixels as standard two-dimensional RGB still images. For the analysis, images were downscaled to each model’s default input resolution. The distance from the camera to the participant was set at 2.0 m for the WBLT and 2.5 m for both the AKET and MTT to ensure full-body capture. The camera height was set at the knee level for the MTT and hip level for both the WBLT and AKET. For an accurate 2D analysis, the camera was positioned perpendicular to the participants and parallel to the plane of movement.

### 2.3. Flexibility and ROM Assessment

All measurements were performed by an experienced physiotherapist. The AKET was used as a key indicator of hamstring flexibility (Figure 1) [13]. The MTT was used to evaluate rectus femoris muscle flexibility [14]. The ankle dorsiflexion ROM was evaluated using the WBLT [16]. For the AKET and MTT, knee joint angles measured using a goniometer were used as reference measurements for comparison, with the greater trochanter, lateral femoral epicondyle, and lateral malleolus serving as anatomical landmarks (Figure 1). For the WBLT, the ankle dorsiflexion angle was assessed using two conventional methods: the trigonometric method and a digital inclinometer (DL164V; STS Co., Ltd., Nagoya, Japan). The digital inclinometer served as a second conventional method commonly used for the WBLT and was placed 15 cm distal to the tibial tuberosity along the anterior tibia to measure the forward inclination angle of the shank relative to the vertical line [23]. The trigonometric method was used as the reference for WBLT validation because it demonstrated the smallest measurement error among the conventional methods in a previous study using the following formula [16,24] (Figure 1):

(1) $$Angle\left(WBLT\right)=90{}^{\circ}-\arctan(\frac{ground-knee\;distance}{wall-heel\;distance})$$

This served as a second conventional method commonly used for the WBLT.

For all three tests, the same images captured for the AI pose-estimation analysis were also analyzed using ImageJ software (1.54g, National Institutes of Health, Bethesda, MD, USA). The same physiotherapist who performed the goniometer and trigonometric measurements identified the same anatomical landmarks on these images to calculate the joint angles as a human-based annotation method. To minimize measurement bias, the examiner performed ImageJ analysis more than one month after completing conventional measurements.

### 2.4. AI Pose-Estimation Measurement

**Figure 2:**
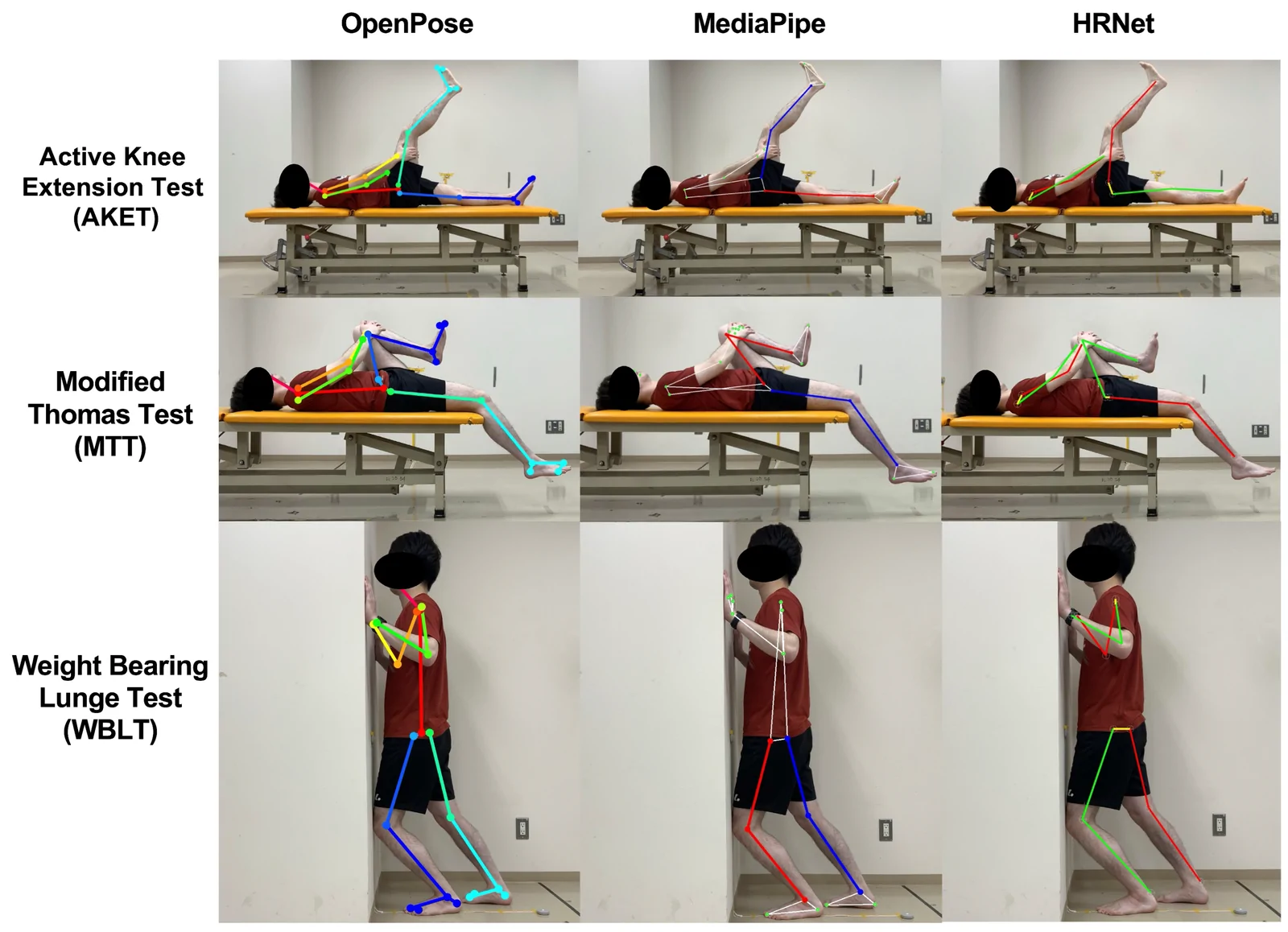
Representative pose estimation outputs for each of the three pose estimation models (OpenPose, MediaPipe, and HRNet), shown for each lower-limb flexibility and ROM test: active knee extension test (AKET), weight-bearing lunge test (WBLT), and modified Thomas test (MTT). OpenPose: right lower limb (green and light blue lines) and left lower limb (blue line). MediaPipe: right lower limb (blue line) and left lower limb (red line). HRNet: right lower limb (red line) and left lower limb (green line). Other colored lines/markers (e.g., yellow, orange) correspond to upper body or trunk landmarks that were not included in the analysis.

Three pose estimation algorithms, MediaPipe [17], OpenPose [18], and HRNet [19] were used (Figure 2). MediaPipe (v0.10.21; BlazePose), provided by Google, was configured to the highest complexity setting using officially distributed pretrained weights [17]. The confidence threshold was set to 0.5 (default), and all other parameters were maintained at their default values. OpenPose (v1.7.0), developed by Carnegie Mellon University, was run in the CPU-only mode and accessed through the Python API (3.10.12) (pyopenpose) [18]. The BODY_25 model with officially distributed pretrained weights was used, and the face and hand modules were disabled. The confidence threshold was set to zero, and all other parameters were maintained at their default values. HRNet was implemented using MATLAB (R2025b, MathWorks, Inc., Natick, MA, USA) [19]. The HRNet-W48 network (“human-full-body-w48”), trained on the COCO keypoint detection dataset, was used. Body regions were detected using a pre-trained YOLOv4 object detector and passed to the keypoint detector. The confidence threshold for valid keypoints was set to 0.2 (default), and all other parameters were set to their default values.

The joint angles were calculated from three extracted landmark coordinates, A = [x_A, y_A], B = [x_B, y_B], and C = [x_C, y_C], with point B as the vertex. Vectors BA = A − B and BC = C − B were defined, and the angle between them was computed in degrees using the dot product. For the AKET and MTT, the knee joint angle was determined from the hip, knee, and ankle joints as the angle between the thigh and shank segments. For the WBLT, the ankle angle was calculated using the knee and ankle joints with a virtual vertical reference point defined above the ankle as the angle between the vertical vector and the shank segment.

**Figure 3:**
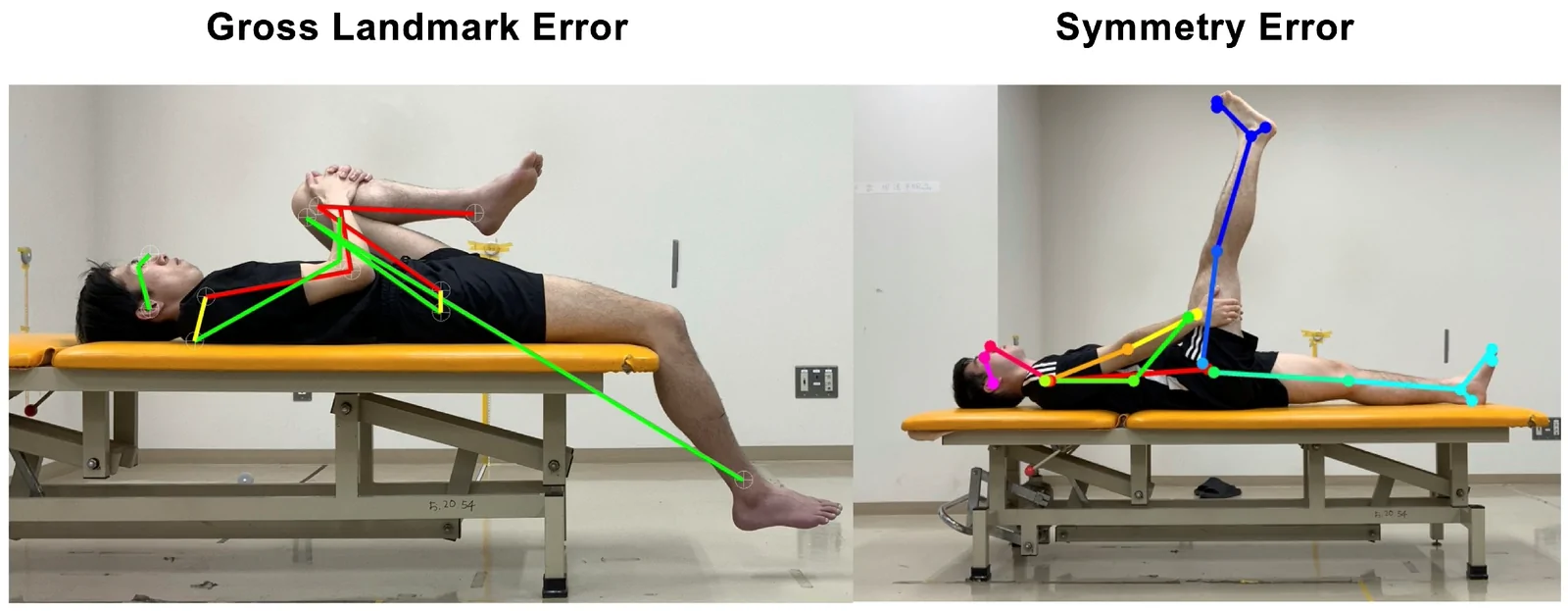
Examples of two failure modes in pose estimation: gross landmark error (**left**, HRNet) and symmetry error (**right**, OpenPose). The gross landmark error was defined as keypoint detection failure, including both the absence of a detected landmark and displacement from the intended anatomical location. The symmetry error was defined as left–right key point misalignment, in which the model placed a landmark on the anatomically opposite side of the body. OpenPose: right lower limb (green and light blue lines) and left lower limb (blue line). HRNet: right lower limb (red line) and left lower limb (green line). Other colored lines/markers (e.g., yellow, orange) correspond to upper body or trunk landmarks that were not included in the analysis.

Estimated points that deviate substantially from the anatomical landmarks reflect a breakdown in detection during pose estimation, and joint angles calculated from such points may substantially distort the estimates of reliability and validity. Therefore, we classified all measurement trials into successful trials and two types of failure (Figure 3), used only the successful trials for analysis, and separately reported the success rate for each test and each model for joint interpretation (Table 1).

The symmetry error was defined as a visually confirmed left–right key point misassignment in which the model placed a landmark on an anatomically opposite side of the body. The gross landmark error was defined as a visually confirmed keypoint detection failure: the absence of a landmark, false detection of background/unrelated objects, or incorrect assignment to an adjacent joint. Only trials without errors were considered successful and retained for the analysis. The success rate was calculated as the percentage of trials classified as successful relative to the total number of measurement attempts for each test and pose-estimation model. The classification was independently validated by an experienced physiotherapist.

### 2.5. Statistical Analysis

Success rates were reported descriptively to characterize how often each model succeeded or failed to detect joints in each test, as this study aimed to evaluate and compare the reliability and validity of pose estimation models across tests. Intra-rater reliability was assessed using the intraclass correlation coefficient (ICC) based on a two-way mixed-effects model, absolute agreement, and single-measure ICC (3, 1) [25]. Participants’ data with missing values due to failure cases, symmetry error, and gross landmark error in trials were excluded from the ICC calculation due to limitations of the statistical software (irr package in R), which requires complete datasets for ICC estimation. The ICC values were interpreted based on the 95% confidence interval (CI): <0.5 (poor), <0.75 (moderate), <0.90 (good), and ≥0.90 (excellent) [25]. We also calculated the standard error of measurement (SEM = SD × √(1 − ICC)) and minimal detectable change (MDC = 1.96 × √2 × SEM) to quantify the absolute reliability and measurement error [26]. Linear regression and Bland–Altman analyses were performed to confirm the validity and agreement between the pose estimation models and conventional methods. Systematic bias and 95% limits of agreement (LOA) were calculated for the differences between the conventional method and both the human-based method (ImageJ) and pose estimation models [27]. When the 95% CI of the mean difference included zero, it was interpreted as indicating no systematic bias in the ICC. Proportional bias was assessed using linear regression analysis, with a significance level of 0.05 [27].

To explore the source of the systematic bias between the AI-based and trigonometric ankle dorsiflexion angles, we performed a post-hoc linear regression analysis in which the conventional ground–knee and heel–wall distances (cm) were used as the independent variable and the corresponding pose-estimation vertical and horizontal shank vector components (pixels) were used as the dependent variable. This analysis was restricted to the same successfully detected trials used in the primary WBLT analyses.

All statistical analyses were performed using R software (version 4.3.1; R Foundation for Statistical Computing, Vienna, Austria). The irr package was used to calculate the intraclass correlation coefficients. The level of significance was set at α = 0.05.

## 3. Results

### 3.1. Success and Failure Counts

The success and failure counts of the three pose estimation models (MediaPipe, OpenPose, and HRNet) during the clinical flexibility tests are presented in Table 1. The success rates of the three models varied across the tests. In the AKET, HRNet achieved the highest success rate (99%), followed by OpenPose (94%) and MediaPipe (94%). In the MTT, OpenPose reached 100%, MediaPipe 98%, and HRNet dropped to 87%, its lowest performance. In the WBLT, both OpenPose and HRNet achieved perfect success rates, whereas MediaPipe recorded a 97% success rate only.

### 3.2. Reliability

Table 2 summarizes the reliability and measurement errors associated with each method. All AKET measurements demonstrated excellent reliability, except for MediaPipe, which was classified as good (95% CI: 0.89–0.96). The SEMs were all within ±5°. The MDC95 was 9.8–10.8° for the pose estimation models, whereas it was ≤5° for the goniometer and ImageJ. In the MTT, the reliability was excellent for all methods except for HRNet (95% CI: 0.83–0.95). MediaPipe and OpenPose showed SEMs of 1.8° and 1.7° and MDC95 values of 5.0° and 4.8°, respectively, whereas the goniometer showed an SEM of 1.6° and an MDC95 of 4.5°. For WBLT, the reliability was excellent for all methods except HRNet (95% CI: 0.87–0.95). All pose estimation models had SEMs and MDC95 values below 2° and 5°, respectively.

### 3.3. Validity

**Figure 4:**
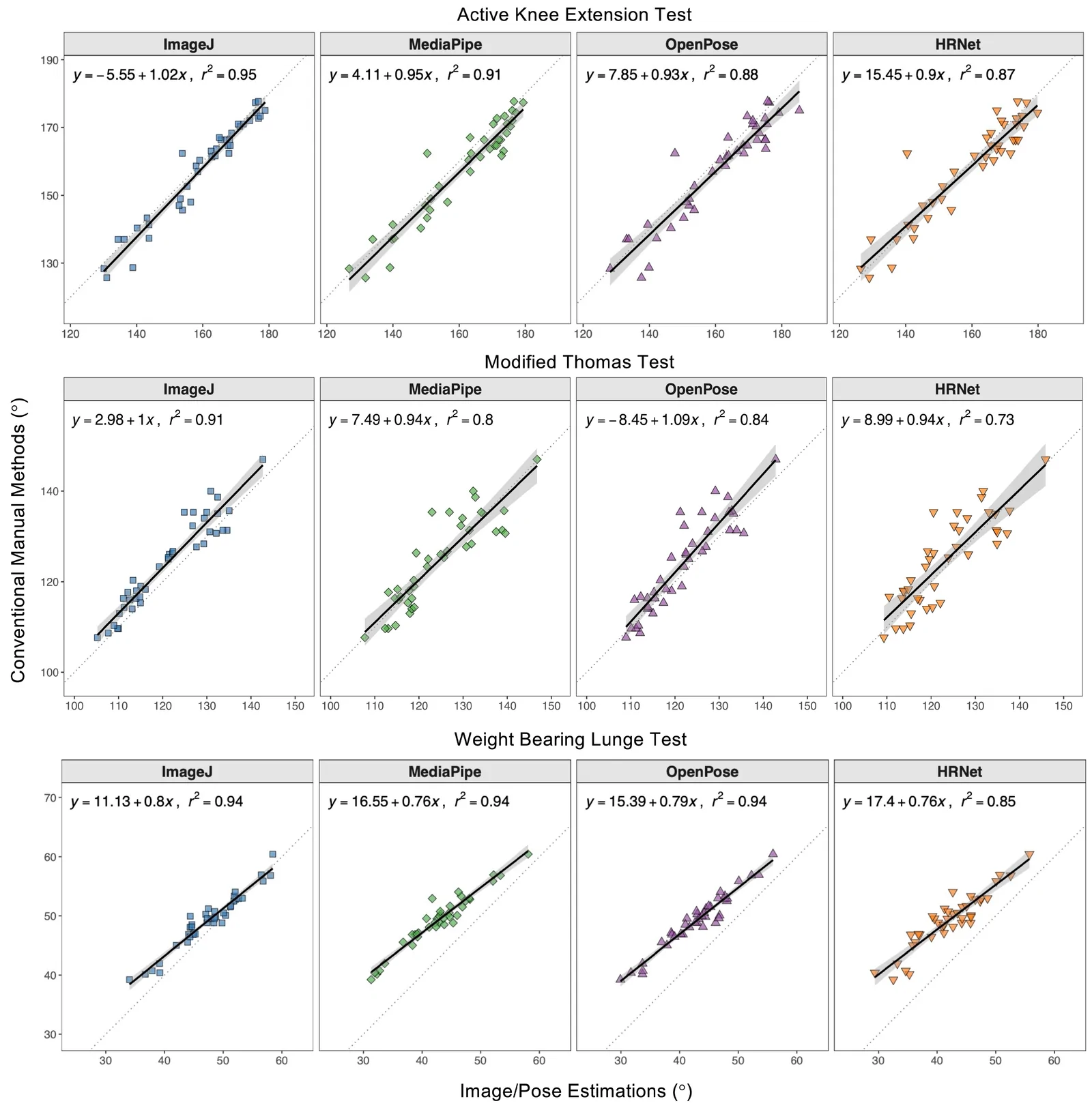
Regression analysis between conventional manual methods (universal goniometer, inclinometer) and digital tools. The solid lines represents linear regression fits, with shaded areas indicating 95% confidence intervals. The dotted lines in the subplots are reference lines with a slope of unity and an intercept of zero.

Concurrent validity and agreement were evaluated using linear regression and Bland–Altman analyses (Figure 4 and Table 3). Linear regression analysis showed high coefficients of determination ranging from 0.73 to 0.94 for all the flexibility tests. In the AKET, Image J, OpenPose, and MediaPipe exhibited significant fixed biases (1.9–3.2°), whereas HRNet did not. No proportional bias was observed. In the MTT, ImageJ and OpenPose demonstrated significant fixed biases (−3.1° and −2.2°, respectively), whereas MediaPipe and HRNet did not demonstrate such biases. Only OpenPose showed a significant proportional bias. In the WBLT, all methods exhibited systematic bias, with an underestimation observed in all AI pose estimation models (−6.3° to −7.2°). Proportional bias was observed in all methods.

As an additional post-hoc analysis, linear regression was performed to examine the agreement of pose-estimation vertical and horizontal shank components and ground-knee and heel-wall distances underlying the trigonometric formula (Table 4). High coefficients of determination were found for both the vertical and horizontal components (R^2^ = 0.785 to 0.868). For the vertical component, across all pose estimation models, no significant intercept was found. In contrast, the horizontal component exhibited a significantly negative intercept across all models (*p* < 0.001).

## 4. Discussion

We hypothesized that pose-estimation models would show acceptable reliability and validity for lower-limb flexibility and ROM assessment, although their performance and agreement with conventional methods may vary across tests.

All models demonstrated high intra-rater reliability, whereas the SEM and MDC_95_ varied depending on the test type and the model. Furthermore, strong correlations with conventional methods were observed, suggesting acceptable validity, whereas fixed and proportional biases were identified for some of the methods. The present findings partially support this hypothesis. These findings support the applicability of pose-estimation models as scalable tools for lower-limb flexibility assessment, although direct interchangeability with conventional methods and interpretation of observed changes require careful consideration due to systematic bias and MDC_95_ values.

### 4.1. Success Rate

The number of successfully detected skeletal joints varied depending on the test type and AI model used. Among the evaluated models, MediaPipe had the lowest overall success rate. This finding is consistent with previous studies reporting that MediaPipe has lower skeletal detection accuracy than OpenPose and HRNet [28]. HRNet achieved nearly perfect success rates in the AKET and WBLT; however, its performance in the MTT decreased to 87%, which was lower than that of the other models. It should be noted that this reduced the amount of analyzable data, which can affect the precision of the estimates, and the ICC and r^2^ require careful interpretation, as the non-random missingness of the trials may have restricted between-subject variance.

In the MTT, HRNet misclassified the left and right body parts and incorrectly mapped the symmetric joints to the same side. This issue can be attributed to the inherent locality of convolutional neural networks (CNNs), which limits their ability to capture global spatial dependencies and long-range interactions [29]. Consequently, in poses such as the MTT, where deep hip flexion causes joints to cluster densely, relying solely on local visual features fails to provide sufficient contextual information, resulting in joint misidentification. Future studies could explore Transformer-based pose estimation models which leverage self-attention mechanisms to effectively capture global context and long-range joint relationships [30,31], potentially overcoming this limitation.

### 4.2. Reliability

Although high intra-rater reliability was observed for all models, measurement error should also be considered in relation to the magnitude of the changes intended to be detected. The AI pose estimation methods demonstrated good to excellent intra-rater reliability, which was broadly comparable to conventional manual methods in all tests. Regarding measurement error, previous studies have reported an MDC_95_ of 12° for the AKET using a goniometer [32], suggesting that the error of AI pose estimation methods may be comparable to that of conventional assessment, with a difference of approximately 5° in MDC_95_. For MTT, MediaPipe and OpenPose demonstrated SEM and MDC_95_ values comparable to those of the goniometer (Table 2). HRNet showed a relatively poorer performance, with an approximately 3° difference compared to the other methods. However, the overall performance of pose estimation was favorable, considering the previously reported SEM value of 6° for the MTT [14]. For WBLT, the AI-based methods showed MDC_95_ values ranging from 2.8° to 4.6°, which were slightly higher than the 1.5° obtained using the trigonometric method in the present study. Nevertheless, these values were comparable to or lower than the previously reported MDC_95_ values of 3.26° for the trigonometric method and 6.02° for the inclinometer measurement [17]. Overall, these findings suggest that pose-estimation methods can be used as a reliable alternative.

### 4.3. Validity

For concurrent validity, linear regression analyses showed high coefficients of determination between all pose estimation methods and the conventional method (Figure 4). Bland–Altman analysis revealed fixed and proportional biases, along with relatively wide LOA (Table 3). For the AKET, ImageJ, MediaPipe, and OpenPose showed small but significant fixed biases ranging from 1.9° to 3.2°, whereas HRNet showed no fixed bias. The half-widths of the LOA were approximately 10°, which was comparable to both the MDC_95_ values in the current study and previously reported AKET measurement error values of 7–12° [30,31]. Therefore, changes within this range may reflect methodological measurement errors rather than true changes in flexibility, warranting caution when using these methods interchangeably. Similar trends were observed for MTT. Significant fixed bias was observed for ImageJ and OpenPose, whereas MediaPipe and HRNet showed no such biases. The LOA half-widths in the AI-based methods were approximately 8–10°, suggesting the need for caution when using these methods interchangeably.

For WBLT, the AI models showed relatively small LOA half-widths ranging from ±3.26° to ±4.42°, but fixed biases exceeding −6° indicated systematic differences from the trigonometric method. To further explore the source of the fixed bias, a post hoc component-wise regression analysis was conducted, comparing the pose estimation-derived vertical and horizontal shank vector components with the ground–knee and heel–wall distances used in the trigonometric calculation (Table 4). The results indicated that a systematic offset between the two methods was primarily observed in the horizontal component, whereas no statistical difference was detected in the vertical component, suggesting that the horizontal component contributed substantially to the observed fixed bias. This can be attributed to differences in landmark definitions used for angle calculations. Specifically, in the horizontal plane, the heel-wall distance includes the distance from the wall to the knee plus the additional distance from the ankle to the posterior edge of the heel. Furthermore, the observed proportional bias may be partly explained by changes in the knee-wall contact point and joint position during deep dorsiflexion and knee flexion. These changes can reduce the discrepancy between the coordinates estimated by the AI and the reference points used in the trigonometric method. It should be noted that the pose estimation coordinates were not calibrated to real-world units; thus, the regression intercepts do not represent a physically interpretable offset in centimeters. A non-zero intercept was interpreted only as a qualitative indication of a consistent discrepancy between the pose estimation models and trigonometric measurements.

Although bias was observed across all tests, these likely reflect differences in landmark definitions, measurement axes, and principles. Therefore, caution is required when comparing AI-based pose estimation measurements with conventional measurements, for example, when applying the same cutoff values or interpretations. However, the high reliability and regression coefficients support the utility of pose estimation models as independent quantitative assessment tools. As pose estimation models are becoming more widely adopted, AI-specific reference or cutoff values may need to be established.

### 4.4. Model Comparisons

Regarding model performance, MediaPipe and OpenPose showed reliability values generally comparable to those obtained using ImageJ (human-based annotation) (Table 2). This suggests that these AI-based methods can replace time-consuming manual annotations in flexibility assessments. HRNet exhibited lower reliability, a larger measurement error, and poorer validity than the other models, despite previous studies reporting its high performance on standard human pose estimation benchmarks, including general adult pose estimation and, more recently, infant pose estimation [19,28]. This discrepancy may reflect reduced robustness for test-specific postures involving complex joint positions; indeed, even studies reporting generally favorable HRNet performance have noted specific failure cases involving such postures (e.g., crossed legs), which fall outside the range of postures represented in standard training data [28]. Therefore, MediaPipe and OpenPose may be more suitable than HRNet for the three flexibility tests conducted in the present study. In particular, MediaPipe has practical advantages for clinical use because it enables rapid joint estimation on mobile devices and central processing units (CPUs) [17].

### 4.5. Limitations

This study has several limitations. First, regarding sample size and statistical power, the sample consisted of 40 lower limbs from 20 participants, and observations within the same participant were not independent. No a priori sample size calculation was performed for the Bland–Altman agreement analysis, and a post-hoc evaluation indicated insufficient statistical power [33]. Moreover, our analysis only included successfully detected cases, leading to a best-case estimation of the algorithms’ true reliability and validity. Consequently, the small sample size, the sample dependency, and the exclusion of some trials may have affected the estimates of reliability and validity.

Second, several aspects of the experimental environment and population were controlled, and the experimental design had a limitation regarding randomization. Measurements were conducted in a well-lit indoor space, meaning the results may not fully translate to outdoor or variable field conditions. In addition, the sample was homogeneous, comprising 20 healthy young adults, which may limit the generalizability of the findings to other populations such as older adults, children, or individuals with musculoskeletal impairments. Furthermore, the order of the flexibility and ROM tests was not randomized, which leaves a potential for carryover effects, such as fatigue or neuromuscular adaptation. However, this risk was partially mitigated by randomizing the measurement methods for each specific test.

Third, there are limitations regarding the reference standards and data analysis. The goniometric and trigonometric methods used as references are subject to human measurement error. Intra-rater reliability was evaluated by a single examiner, and inter-rater reliability was not assessed.

Finally, this study focused on widely used and practically feasible AI-based pose estimation algorithms rather than evaluating the entire spectrum of available models. Recent Transformer-based models, such as TokenPose [31] and ViTPose [30], have demonstrated competitive or superior performance on standard benchmarks compared to representative CNN-based methods [28]. Future studies should investigate whether these advanced models yield improved accuracy in musculoskeletal flexibility and joint ROM assessments.

## 5. Conclusions

This study demonstrated that for assessing lower-limb flexibility and ROM, AI-based pose estimation models showed good to excellent reliability and supported concurrent validity with conventional methods, although a systematic bias was observed. Clinicians should consider measurement errors, particularly the MDC95 for the AKET, when interpreting observed changes. OpenPose and MediaPipe demonstrated relatively better performance than HRNet. Although careful consideration is required regarding interchangeability with conventional methods, AI-based pose estimation can serve as an efficient and scalable assessment tool for evaluating lower-limb flexibility and ROM.

## Figures and Tables

**Table 1:** Successes and errors in the trials for each pose-estimation model.

Method	Total Trials	Success Rate (%)	Gross Landmark Error	Symmetrical Error
**AKET**				
MediaPipe	120	113 (94%)	4	3
OpenPose	120	113 (94%)	2	5
HRNet	120	119 (99%)	0	1
**MTT**				
MediaPipe	120	117 (98%)	0	3
OpenPose	120	120 (100%)	0	0
HRNet	120	104 (87%)	11	5
**WBLT**				
MediaPipe	120	116 (97%)	4	0
OpenPose	120	120 (100%)	0	0
HRNet	120	120 (100%)	0	0

Data are reported as numbers (%). Success was defined as trials without errors. Gross landmark error refers to the exclusion of trials because of significant anatomical misplacement of key points. Symmetry errors occurred when the models misidentified contralateral limbs as target limbs. Successes in the analyzed trials represented the final number of valid trials included in the statistical analysis. AKET: active knee extension test; MTT: modified Thomas test; WBLT: weight-bearing lunge test.

**Table 2:** Measurement reliability results for each pose-estimation model.

Methods	Mean (SD) (°)	ICC (3, 1) (95% CI)	SEM (°)	MDC_95_ (°)
**AKET**				
Goniometer	157 (14.6)	0.98 (0.97, 0.99)	1.8	5.0
ImageJ	159 (13.9)	0.98 (0.97, 0.99)	1.7	4.9
MediaPipe	160 (15.2)	0.94 (0.89, 0.96)	3.9	10.7
OpenPose	161 (14.5)	0.94 (0.90, 0.97)	3.5	9.8
HRNet	159 (15.6)	0.94 (0.90, 0.96)	3.9	10.8
**MTT**				
Goniometer	124 (10.1)	0.97 (0.95, 0.99)	1.6	4.5
ImageJ	121 (9.6)	0.96 (0.93, 0.98)	2.0	5.6
MediaPipe	124 (9.4)	0.96 (0.94, 0.98)	1.8	5.0
OpenPose	122 (8.5)	0.96 (0.93, 0.98)	1.7	4.8
HRNet	123 (9.1)	0.90 (0.83, 0.95)	2.8	7.8
**WBLT**				
Trigonometric method	49 (4.7)	0.99 (0.98, 0.99)	0.6	1.5
Inclinometer	54 (6.6)	0.99 (0.97, 0.99)	0.8	2.2
ImageJ	48 (5.6)	0.96 (0.93, 0.98)	1.1	3.1
MediaPipe	43 (5.8)	0.96 (0.93, 0.98)	1.1	3.2
OpenPose	43 (5.8)	0.97 (0.95, 0.98)	1.0	2.8
HRNet	42 (5.8)	0.92 (0.87, 0.95)	1.7	4.6

AKET: active knee extension test; MTT: modified Thomas test; WBLT: weight-bearing lunge test; SD: standard deviation; ICC: intraclass correlation coefficient; CI: confidence interval; SEM: standard error of measurement; MDC: minimal detectable change.

**Table 3:** Bland–Altman analysis results of pose estimation models and conventional manual measurements.

Methods	Bias (95% CI)	LOA (Lower, Upper)	Proportional Bias (*p*-Value)
**AKET**			
ImageJ	1.85 (0.76, 2.94)	±6.69 (−4.83, 8.54)	0.19
MediaPipe	3.23 (1.78, 4.67)	±8.73 (−5.50, 11.96)	0.99
OpenPose	3.03 (1.40, 4.66)	±10.02 (−6.99, 13.05)	0.87
HRNet	1.15 (−0.63, 2.92)	±10.87 (−9.72, 12.01)	0.49
**MTT**			
ImageJ	−3.06 (−4.02, −2.09)	±5.92 (−8.97, 2.86)	0.34
MediaPipe	−0.14 (−1.59, 1.32)	±8.80 (−8.93, 8.66)	0.48
OpenPose	−2.23 (−3.54, −0.92)	±8.04 (−10.27, 5.81)	0.01
HRNet	−1.32 (−2.99, 0.37)	±10.18 (−11.50, 8.86)	0.27
**WBLT**			
ImageJ	−1.73 (−2.24, −1.22)	±3.13 (−4.86, 1.40)	<0.001
MediaPipe	−6.49 (−7.09, −5.89)	±3.63 (−10.12, −2.86)	<0.001
OpenPose	−6.25 (−7.09, −5.72)	±3.25 (−9.51, −3.00)	<0.001
HRNet	−7.17 (−7.89, −6.45)	±4.42 (−11.59, −2.75)	<0.001

Bias represents the mean difference between methods. Proportional bias was assessed using linear regression of the difference against the mean, with statistical significance set at *p* < 0.05. AKET: active knee extension test; MTT: modified Thomas test; WBLT: weight-bearing lunge test; CI: confidence interval; LOA: limits of agreement.

**Table 4:** Linear regression of the pose-estimation vertical and horizontal shank vector components and ground–knee and heel–wall distances used for the trigonometric method.

Models	Intercept (95% CI)	*p*-Value (Intercept)	Estimate (95% CI)	r^2^
**HRNet**				
Ground-knee distance	25.5 (−8.3, 59.4)	0.138	13.01 (11.96, 14.05)	0.843
Heel-wall distance	−77.6 (−124.4, −30.7)	0.001	12.90 (11.64, 14.15)	0.785
**OpenPose**				
Ground-knee distance	6.3 (−25.0, 37.7)	0.689	13.35 (12.38, 14.32)	0.868
Heel-wall distance	−97.6 (−131.9, −63.3)	<0.001	13.57 (12.65, 14.49)	0.883
**MediaPipe**				
Ground-knee distance	15.8 (−13.9, 45.6)	0.293	12.67 (11.75, 13.59)	0.868
Heel-wall distance	−67.2 (−106.4, −28.0)	<0.001	12.33 (11.28, 13.39)	0.827

Linear regression was conducted to explore the source of systematic angular bias in the WBLT. The vertical and horizontal shank vector components from pose estimation were regressed against the ground–knee and heel–wall distances used for the trigonometric method, respectively. An intercept significantly different from zero (*p* < 0.05, with the 95% CI excluding zero) indicates a systematic offset. CI: confidence interval.

## Data Availability

The data presented in this study are available on request from the corresponding author due to privacy/ethical restrictions.

## Abbreviations

The following abbreviations are used in this manuscript.

AKET	Active Knee Extension Test
MTT	Modified Thomas Test
WBLT	Weight-Bearing Lunge Test
ICC	Intraclass Correlation Coefficient
SEM	Standard Error of Measurement
MDC	Minimal Detectable Change
LOA	Limits of Agreement
CI	Confidence Interval
ROM	Range of Motion
CNN	Convolutional Neural Network

## References

[1] Witvrouw E., Danneels L., Asselman P., D’Have T., Cambier D. (2003). Muscle Flexibility as a Risk Factor for Developing Muscle Injuries in Male Professional Soccer Players: A Prospective Study. Am. J. Sports Med..

[2] De Vos R.-J., Reurink G., Goudswaard G.-J., Moen M.H., Weir A., Tol J.L. (2014). Clinical Findings Just after Return to Play Predict Hamstring Re-Injury, but Baseline MRI Findings Do Not. Br. J. Sports Med..

[3] Hoch M.C., Farwell K.E., Gaven S.L., Weinhandl J.T. (2015). Weight-Bearing Dorsiflexion Range of Motion and Landing Biomechanics in Individuals with Chronic Ankle Instability. J. Athl. Train..

[4] Takei S., Torii S., Taketomi S., Iizuka S., Tojima M., Iwanuma S., Iida Y., Tanaka S. (2023). Developmental Stage and Lower Quadriceps Flexibilities and Decreased Gastrocnemius Flexibilities Are Predictive Risk Factors for Developing Osgood–Schlatter Disease in Adolescent Male Soccer Players. Knee Surg. Sports Traumatol. Arthrosc..

[5] Zein M.I., Mokkenstorm M.J.K., Cardinale M., Holtzhausen L., Whiteley R., Moen M.H., Reurink G., Tol J.L. (2024). Baseline Clinical and MRI Risk Factors for Hamstring Reinjury Showing the Value of Performing Baseline MRI and Delaying Return to Play: A Multicentre, Prospective Cohort of 330 Acute Hamstring Injuries. Br. J. Sports Med..

[6] Chung J.-L., Ong L.-Y., Leow M.-C. (2022). Comparative Analysis of Skeleton-Based Human Pose Estimation. Future Internet.

[7] Latreche A., Kelaiaia R., Chemori A., Kerboua A. (2023). Reliability and Validity Analysis of MediaPipe-Based Measurement System for Some Human Rehabilitation Motions. Measurement.

[8] Dibenedetto G., Sotiropoulos S., Polignano M., Cavallo G., Lops P. (2025). Comparing Human Pose Estimation through Deep Learning Approaches: An Overview. Comput. Vis. Image Underst..

[9] Ino T., Samukawa M., Ishida T., Wada N., Koshino Y., Kasahara S., Tohyama H. (2023). Validity of AI-Based Gait Analysis for Simultaneous Measurement of Bilateral Lower Limb Kinematics Using a Single Video Camera. Sensors.

[10] Washabaugh E.P., Shanmugam T.A., Ranganathan R., Krishnan C. (2022). Comparing the Accuracy of Open-Source Pose Estimation Methods for Measuring Gait Kinematics. Gait Posture.

[11] Yoma M., Llurda-Almuzara L., Herrington L., Jones R. (2025). Reliability and Validity of Lower Extremity and Trunk Kinematics Measured with Markerless Motion Capture during Sports-Related and Functional Tasks: A Systematic Review. J. Sports Sci..

[12] Ota M., Tateuchi H., Hashiguchi T., Kato T., Ogino Y., Yamagata M., Ichihashi N. (2020). Verification of Reliability and Validity of Motion Analysis Systems during Bilateral Squat Using Human Pose Tracking Algorithm. Gait Posture.

[13] Hamid M.S.A., Ali M.R.M., Yusof A. (2013). Interrater and Intrarater Reliability of the Active Knee Extension (AKE) Test among Healthy Adults. J. Phys. Ther. Sci..

[14] Peeler J.D., Anderson J.E. (2008). Reliability Limits Of The Modified Thomas Test For Assessing Rectus Femoris Muscle Flexibility About The Knee Joint. J. Athl. Train..

[15] Peeler J., Leiter J. (2013). Using Digital Photography to Document Rectus Femoris Flexibility: A Reliability Study of the Modified Thomas Test. Physiother. Theory Pract..

[16] Langarika-Rocafort A., Emparanza J.I., Aramendi J.F., Castellano J., Calleja-González J. (2017). Intra-Rater Reliability and Agreement of Various Methods of Measurement to Assess Dorsiflexion in the Weight Bearing Dorsiflexion Lunge Test (WBLT) among Female Athletes. Phys. Ther. Sport.

[17] Bazarevsky V., Grishchenko I., Raveendran K., Zhu T., Zhang F., Grundmann M. (2020). BlazePose: On-Device Real-Time Body Pose Tracking. arXiv.

[18] Cao Z., Hidalgo G., Simon T., Wei S.-E., Sheikh Y. (2021). OpenPose: Realtime Multi-Person 2D Pose Estimation Using Part Affinity Fields. IEEE Trans. Pattern Anal. Mach. Intell..

[19] Wang J., Sun K., Cheng T., Jiang B., Deng C., Zhao Y., Liu D., Mu Y., Tan M., Wang X. (2021). Deep High-Resolution Representation Learning for Visual Recognition. IEEE Trans. Pattern Anal. Mach. Intell..

[20] Bennell K., Talbot R., Wajswelner H., Techovanich W., Kelly D., Hall A. (1998). Intra-Rater and Inter-Rater Reliability of a Weight-Bearing Lunge Measure of Ankle Dorsiflexion. Aust. J. Physiother..

[21] Walter S.D., Eliasziw M., Donner A. (1998). Sample Size and Optimal Designs for Reliability Studies. Stat. Med..

[22] Arnold J.E., Camus M.S., Freeman K.P., Giori L., Hooijberg E.H., Jeffery U., Korchia J., Meindel M.J., Moore A.R., Sisson S.C. (2019). ASVCP Guidelines: Principles of Quality Assurance and Standards for Veterinary Clinical Pathology (Version 3.0): Developed by the American Society for Veterinary Clinical Pathology’s (ASVCP) Quality Assurance and Laboratory Standards (QALS) Committee. Vet. Clin. Pathol..

[23] Hall E.A., Docherty C.L. (2017). Validity of Clinical Outcome Measures to Evaluate Ankle Range of Motion during the Weight-Bearing Lunge Test. J. Sci. Med. Sport.

[24] Howe L.P., Bampouras T.M., North J.S., Waldron M. (2020). Within-Session Reliability for Inter-Limb Asymmetries in Ankle Dorsiflexion Range of Motion Measured During the Weight-Bearing Lunge Test. Int. J. Sports Phys. Ther..

[25] Koo T.K., Li M.Y. (2016). A Guideline of Selecting and Reporting Intraclass Correlation Coefficients for Reliability Research. J. Chiropr. Med..

[26] Weir J.P. (2005). Quantifying test-retest reliability using the intraclass correlation coefficient and the SEM. J. Strength Cond. Res..

[27] Giavarina D. (2015). Understanding Bland Altman Analysis. Biochem. Med..

[28] Gama F., Mísař M., Navara L., Popescu S.T., Hoffmann M. (2025). Automatic Infant 2D Pose Estimation from Videos: Comparing Seven Deep Neural Network Methods. Behav. Res..

[29] Yang S., Quan Z., Nie M., Yang W. (2021). TransPose: Keypoint Localization via Transformer. Proceedings of the 2021 IEEE/CVF International Conference on Computer Vision (ICCV).

[30] Xu Y., Zhang J., Zhang Q., Tao D. (2022). ViTPose: Simple Vision Transformer Baselines for Human Pose Estimation. arXiv.

[31] Li Y., Zhang S., Wang Z., Yang S., Yang W., Xia S.-T., Zhou E. (2021). TokenPose: Learning Keypoint Tokens for Human Pose Estimation. Proceedings of the 2021 IEEE/CVF International Conference on Computer Vision (ICCV).

[32] Olivencia O., Godinez G.M., Dages J., Duda C., Kaplan K., Kolber M.J. (2020). The reliability and minimal detectable change of the ely and active knee extension tests. Int. J. Sports Phys. Ther..

[33] Lu M.-J., Zhong W.-H., Liu Y.-X., Miao H.-Z., Li Y.-C., Ji M.-H. (2016). Sample Size for Assessing Agreement between Two Methods of Measurement by Bland−Altman Method. Int. J. Biostat..

